# Comorbidity with substance abuse and its influence in a forensic population: A retrospective study

**DOI:** 10.1192/j.eurpsy.2021.345

**Published:** 2021-08-13

**Authors:** M. Jesus

**Affiliations:** Centro De Responsabilidade Integrada De Psiquiatria, Centro Hospitalar e Universitário de Coimbra, Coimbra, Portugal

**Keywords:** substance abuse, ACE, Dual pathology, Forensic

## Abstract

**Introduction:**

The criminality associated with psychiatric disorders has been extensively studied with some studies showing a greater risk of violence in these patients. Substance abuse has been long linked to criminal and antisocial behaviours, but what happens when is in comorbidity with other psychiatric disorders.

**Objectives:**

The authors aim to study the impact of substance abuse comorbidity in type of crime and other characteristics in a forensic ward population.

**Methods:**

A retrospective study was designed, including patients admitted in the Forensic ward of Coimbra Hospital and University Center between 2018 and 2020.

**Results:**

Our study included 110 patients, 39 of which had comorbidity with substance abuse. Although the authors couldn’t find differences in the type of crime committed regarding the patient’s primary diagnosis, substance abuse was significantly associated with non-violent crimes. The prevalence of homicide was significantly inferior in psychoactive substance users and the prevalence of domestic violence was significantly greater. However, the prevalence of a criminal history was significantly higher in patients with comorbidity with substance abuse. Patients with substance abuse had significantly higher childhood adverse events reports.

**Conclusions:**

Interestingly, criminal behaviors prior to admission were more frequent in patients with substance abuse, which is understandable. However, the type of crimes were significantly less serious in this patients, which can mean that, although these consumptions are a risk factor for criminal behavior, the association in less important in crimes like murder. The exposition to childhood adverse events is a well-known risk factor for substance abuse in adulthood.

**Disclosure:**

No significant relationships.

